# Team Science Approaches to Unravel Monogenic Parkinson’s Disease on a Global Scale

**DOI:** 10.1002/mds.29925

**Published:** 2024-07-30

**Authors:** Johanna Junker, Lara M. Lange, Eva-Juliane Vollstedt, Karisha Roopnarain, Maria Leila M. Doquenia, Azlina Ahmad Annuar, Micol Avenali, Soraya Bardien, Natascha Bahr, Melina Ellis, Caterina Galandra, Thomas Gasser, Peter Heutink, Anastasia Illarionova, Yuliia Kanana, Ignacio J. Keller Sarmiento, Kishore R. Kumar, Shen-Yang Lim, Harutyun Madoev, Ignacio F. Mata, Niccolò E. Mencacci, Mike A. Nalls, Shalini Padmanabhan, Cholpon Shambetova, J. C. Solle, Ai-Huey Tan, Joanne Trinh, Enza Maria Valente, Andrew Singleton, Cornelis Blauwendraat, Katja Lohmann, Zih-Hua Fang, Christine Klein, the Global Parkinson’s Genetics Program (GP2)

**Affiliations:** 1 Institute of Neurogenetics, University of Luebeck, Luebeck, Germany; 2 Department of Neurology, University Clinic Schleswig-Holstein, Luebeck, Germany; 3 Department of Neurology, University of Free State, Bloemfontein, South Africa; 4 Department of Biomedical Science, Faculty of Medicine, University of Malaya, Kuala Lumpur, Malaysia; 5 Department of Brain and Behavioral Sciences, University of Pavia, Pavia, Italy; 6 IRCCS Mondino Foundation, Pavia, Italy; 7 Division of Molecular Biology and Human Genetics, Department of Biomedical Sciences, Faculty of Medicine and Health Sciences, Stellenbosch University, Cape Town, South Africa; 8 South African Medical Research Council/Stellenbosch University Genomics of Brain Disorders Research Unit, Stellenbosch University, Cape Town, South Africa; 9 Northcott Neuroscience Laboratory, ANZAC Research Institute, Concord, New South Wales, Australia; 10 Faculty of Medicine and Health, University of Sydney, Sydney, New South Wales, Australia; 11 Department of Molecular Medicine, University of Pavia, Pavia, Italy; 12 Department for Neurodegenerative Diseases, Hertie Institute for Clinical Brain Research, University of Tuebingen, Tuebingen, Germany; 13 German Center for Neurodegenerative Diseases, Tuebingen, Germany; 14 Ken and Ruth Davee Department of Neurology and Simpson Querrey Center for Neurogenetics, Northwestern University, Feinberg School of Medicine, Chicago, Illinois, USA; 15 Translational Neurogenomics, Genomic and Inherited Disease Program, Garvan Institute of Medical Research and UNSW Sydney, Darlinghurst, New South Wales, Australia; 16 Molecular Medicine Laboratory and Neurology Department, Concord Repatriation General Hospital, The University of Sydney, Concord, New South Wales, Australia; 17 Division of Neurology, Department of Medicine, and the Mah Pooi Soo and Tan Chin Nam Centre for Parkinson’s and Related Disorders, Faculty of Medicine, University of Malaya, Kuala Lumpur, Malaysia; 18 Genomic Medicine Institute, Cleveland Clinic, Cleveland, Ohio, USA; 19 DataTecnica, Washington, DC, USA; 20 Center for Alzheimer’s and Related Dementias, National Institute on Aging and National Institute of Neurological Disorders and Stroke, National Institutes of Health, Bethesda, Maryland, USA; 21 Discovery and Translational Research, The Michael J. Fox Foundation for Parkinson’s Research, New York, New York, USA; 22 Department of Clinical Research, The Michael J. Fox Foundation for Parkinson’s Research, New York, New York, USA; 23 Laboratory of Neurogenetics, National Institute on Aging, National Institutes of Health, Bethesda, Maryland, USA

**Keywords:** Parkinson’s disease, GP2, MJFF GMPD, monogenic Parkinson’s disease, parkinsonism

## Abstract

**Background:**

Until recently, about three-quarters of all monogenic Parkinson’s disease (PD) studies were performed in European/White ancestry, thereby severely limiting our insights into genotype–phenotype relationships at a global scale.

**Objective:**

To identify the multi-ancestry spectrum of monogenic PD.

**Methods:**

The first systematic approach to embrace monogenic PD worldwide, The Michael J. Fox Foundation Global Monogenic PD Project, contacted authors of publications reporting individuals carrying pathogenic variants in known PD-causing genes. In contrast, the Global Parkinson’s Genetics Program’s Monogenic Network took a different approach by targeting PD centers underrepresented or not yet represented in the medical literature.

**Results:**

In this article, we describe combining both efforts in a merger project resulting in a global monogenic PD cohort with the buildup of a sustainable infrastructure to identify the multi-ancestry spectrum of monogenic PD and enable studies of factors modifying penetrance and expressivity of monogenic PD.

**Conclusions:**

This effort demonstrates the value of future research based on team science approaches to generate comprehensive and globally relevant results. © 2024 State of New South Wales and The Author(s). *Movement Disorders* published by Wiley Periodicals LLC on behalf of International Parkinson and Movement Disorder Society. This article has been contributed to by U.S. Government employees and their work is in the public domain in the USA.

Although monogenic forms of Parkinson’s disease (PD) have been described worldwide, about three-quarters of all PD genetics studies were performed in European/White populations,^1^ thereby severely limiting our current insight into genotype–phenotype relationships at a global, multi-ancestry scale and contributing further to healthcare and research disparities.^2^ The first systematic approach to embrace monogenic PD worldwide, The Michael J. Fox Foundation Global Monogenic PD (MJFF GMPD) project, was built on the MDSGene Database (https://www.mdsgene.org) that compiles published genotype–phenotype relationships for monogenic PD^1,3–5^ and other movement disorders. Corresponding authors of all included publications were contacted, and individual-level data were collected on almost 4000 individuals from 92 centers in 42 countries, including affected and unaffected carriers of pathogenic variants in genes implicated in monogenic PD (including *SNCA*, *LRRK2*, *VPS35*, *PRKN*, *PINK1*, *PARK7/DJ-1*, and the PD risk gene *GBA1*).^1^ More recently, another global science project was initiated: the Global Parkinson’s Genetics Program (GP2; https://gp2.org). GP2 took a different but complementary approach by specifically addressing centers that did not have access to genetic testing or had not participated in PD research before.^6,7^

In this article, we describe the merger of both efforts and how, by building on The MJFF GMPD project, GP2 successfully expands the global network and data resource for monogenic PD.

## Materials and Methods

The recruitment strategies of MJFF GMPD and the Global Parkinson’s Genetics Program’s Monogenic Network (GP2’s MN) effort were described in detail previously.^1,8,9^ For MJFF GMPD project, individual-level clinical, demographic, and genetic data on affected and unaffected carriers of pathogenic variants in known PD genes (*SNCA*, *LRRK2*, *VPS35*, *PRKN*, *PINK1*, *PARK7*) and the PD risk gene *GBA1* were collected, and participating centers were invited to share DNA samples from submitted individuals.^1^ To identify collaborators for the GP2’s MN, we extended the publication-based approach by including researchers identified through personal contacts, participation in PD consortia, and advertising GP2 at congresses. A main interest of GP2’s MN was to include research centers not identifiable through a publication-based approach. DNA samples and demographic, clinical, and existing genetic data of individuals with an unsolved but suspected monogenic cause of PD based on an early age at onset (AAO; ≤50 years of age) or a positive family history and their affected or unaffected family members were collected.^9^

To facilitate the merger of both projects, collaborators from both efforts were divided into three groups based on their involvement in either both projects (group 1), only in MJFF GMPD (group 2), or only in the GP2’s MN (group 3) project. Participants of The MJFF GMPD project received customized emails including relevant information about GP2 and an online survey gauging their interest in participating and their availability of DNA samples. Collaborators interested in joining GP2 were given standardized instructions for the onboarding process to GP2’s MN.^9^ Further, all collaborators previously involved in The MJFF GMPD project (groups 1 and 2) were asked for their permission to transfer existing data and samples to GP2. In parallel, centers involved in only GP2’s MN (group 3) were asked to also submit samples and data from known carriers of pathogenic variants.

This article includes centers with GP2 compliance approval until November 2023. Compliance approval is based on an eligible consent form enabling international sample and data sharing and approval by the local Ethics Committee.^9^

The assignment to underrepresented countries was based on the World Bank’s classification of income status (https://data.worldbank.org/country/XO), which classifies low- and middle-income countries as underrepresented.

Percentages in the Results section are given as valid percentages.

### Data Sharing

GP2 partnered with the online cloud computing platform Accelerating Medicines Partnership—Parkinson’s Disease (AMP PD; https://amp-pd.org) to share data generated by GP2. Anonymized data can be shared upon request, and qualified researchers are encouraged to apply for direct access to the data through AMP PD.

## Results

**FIG. 1.**
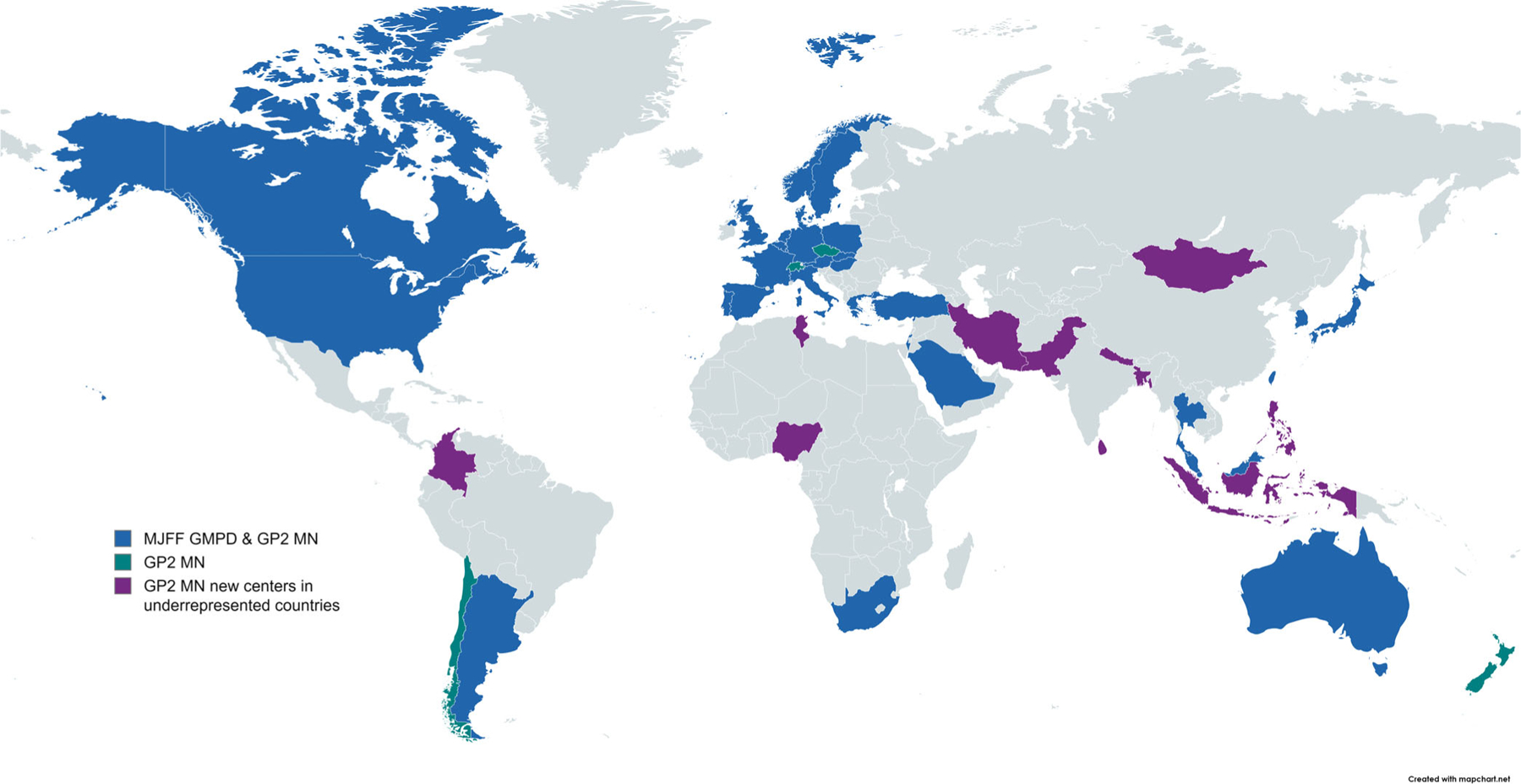
World map of centers participating in the Global Parkinson’s Genetics Program’s Monogenic Network (GP2’s MN) and The Michael J. Fox Foundation Global Monogenic PD (MJFF GMPD) project. Colored are the countries from which one or more centers participated in The MJFF GMPD project and GP2’s MN (blue) or only in GP2’s MN (green). Colored in purple are new centers in underrepresented countries that were recruited based on the new approach of GP2’s MN. Blue: Argentina, Australia, Austria, Belgium, Canada, Denmark, France, Germany, Greece, Hungary, Israel, Italy, Japan, Luxembourg, Malaysia, Netherlands, Norway, Poland, Portugal, Saudi Arabia, Singapore, Slovakia, South Africa, South Korea, Spain, Sweden, Taiwan, Thailand, Turkey, United Kingdom, and United States. Green: Chile, Czechia, New Zealand, and Switzerland. Purple: Bangladesh, Colombia, Indonesia, Iran, Mongolia, Nepal, Nigeria, Pakistan, Philippines, Sri Lanka, and Tunisia.

To date, 100 centers from 46 countries (Fig. 1) are included in GP2’s MN. All centers previously identified through The MJFF GMPD project^1^ were invited to join GP2 as part of the merger, and 38 (41.3%) of these are now participating in GP2’s MN. Reasons for non-participation were diverse, for example, no response to the invitation to participate, delay or failure of the onboarding process because of bureaucratic hurdles (eg, consent forms not meeting the legal requirements of GP2), or inability to provide DNA samples in addition to clinical data. Nineteen (19%) centers from 15 countries included in the GP2’s MN were from formerly underrepresented countries (Fig. 1), 11 countries of which were not previously part of The MJFF GMPD project.

Thirty-nine centers and the PDGene consortium (https://www.parkinson.org/advancing-research/our-research/pdgeneration) already submitted samples and clinical data to GP2’s MN for further analysis. The onboarding process is ongoing for the remaining 60 centers, so samples and data have not yet been shared. To date, 5567 samples have been sent to the coordinating GP2 site in Luebeck, Germany, including 468 samples with pending clinical data. The vast majority (n = 4824) of these 5099 ready-to-analyze samples are from affected individuals with PD/parkinsonism, and only 275 are from unaffected relatives or unaffected pathogenic variant carriers. A subset of these (n = 293) were from individuals previously submitted to The MJFF GMPD project and now transferred to GP2. The comparison of collected demographic and clinical data, as well as the reported genetic findings of the 3185 affected individuals included in the MJFF GMPD, and the newly built GP2’s MN cohort, including 4824 affected individuals, are presented in Table 1. Further, we also present the respective data of a total of 539 affected individuals with reported genetic findings, including the 293 individuals transferred from The MJFF GMPD project and 246 submitted to GP2 after the merger. Heterozygous GBA1 variant carriers (38.4%–51.7%), followed by heterozygous *LRRK2* (24.1%–41.0%) and biallelic *PRKN* (13.5%–14.7%) carriers were most frequently reported among the three groups, whereas variants in the other PD genes were rarer (Table 1).

More than half of the entire GP2 MN cohort had an AAO ≤ 50 years (54.1%; missing data 16.9%). The median AAO was 49 years (interquartile range, 41–62 years). The family history was positive for 59.1% (missing data 22.9%), and for 7.0% (missing data 55.1%) consanguinity was reported. Most of the affected individuals were of Asian (47.6%) or White/European (44.8%) ancestry (missing data 23.3%); other rarer ancestries included Arab (2.8%), Hispanic/Latino (1.9%), Black/African American (0.8%), Ashkenazi Jewish (0.4%), American Indian/Alaska Native (0.1%), Native Hawaiian/Other Pacific Islander (<0.1%), and other/mixed ancestries (1.6%).

## Discussion

To date, 100 centers from 46 countries are included in GP2’s MN, 38% of which were previously recruited through The MJFF GMPD project.^1^ An important reason for other MJFF GMPD centers not to participate in GP2 was that the principal investigator was no longer active, demonstrating the importance of dedicated researchers to drive projects like GP2’s MN. The two projects followed different approaches when recruiting collaborators. The merger of both efforts with complementary approaches enables the comprehensive identification of centers working in the PD genetics field, including not only centers identifiable through publications on PD genetics (MJFF GMPD) but also less visible centers (GP2’s MN), and, further, facilitates the buildup of an inclusive study population with a diverse ancestral background. Sixty-two centers not previously participating in The MJFF GMPD project were recruited for GP2’s MN, including 16 centers from underrepresented countries, underlining the importance of the inclusive GP2 approach. However, the research infrastructure in these countries often posed a challenge to the onboarding process and required intensified efforts and support. Our goal is to overcome these bureaucratic and logistical hurdles to enable global representation of monogenic PD. One way of doing this will be including and supporting established PD consortia in these areas, for example, LARGE-PD (https://large-pd.org/).

So far, a total of ~5600 samples, including corresponding demographic and clinical data from ~5100 affected and unaffected individuals, have been submitted to GP2’s MN, compared with ~3900 affected and unaffected individuals collected through The MJFF GMPD project.^1^ The GP2 recruitment policy enabled an increase in the proportion of non-White/European ancestries among the included individuals from 24% (MJFF GMPD) to 55% (GP2’s MN), which was mainly driven by a substantial increase in individuals of Asian ancestry from 5% (MJFF GMPD) to 48% (GP2’s MN). Reflecting the inclusion criteria of GP2’s MN, a high percentage of affected individuals with a positive family history was achieved (~60%). By allowing GP2 collaborators to submit solved monogenic cases, we expanded the number of self-reported mutation carriers within GP2’s MN cohort to a total of 539 individuals. Not surprisingly, carriers of *GBA1* variants were most frequent (52%), followed by *LRRK2* (24%) and *PRKN* (15%), whereas the remaining monogenic forms were significantly rarer. Notably, the genetic data were reported by collaborators and are based on different analytical methods (eg, Sanger sequencing, gene panel, or exome sequencing), and findings have not yet been completely validated within GP2. Moving forward, all samples submitted to GP2’s MN will undergo genome-wide genotyping with the NeuroBooster array^10^ and short-read whole-genome sequencing, regardless of whether genetic testing was performed before.

In addition to the increase in the number of individuals, including samples and clinical data, and the expansion of ancestral diversity compared with MJFF GMPD, the focus on unsolved cases is a unique attribute of the GP2’s MN. Integrating MJFF GMPD project into GP2 has widened the scope of GP2’s MN. Merging both projects will allow the creation of a sufficient infrastructure to identify the clinical spectrum of monogenic PD across diverse ancestries and also facilitate the investigation of factors modifying monogenic PD by identifying a large number of mutation carriers. Together, the combined effort now allows for a more comprehensive and collaborative approach to understanding monogenic PD at a global scale. Identifying patients with monogenic PD also provides the basis for recruiting individuals with genetic PD/parkinsonism for future gene-specific clinical trials, which is a dedicated aim of one of GP2’s interest groups.

Our efforts of combining two large genetic PD research initiatives underline that the future of research should be based on team science approaches, with an emphasis on including formerly underrepresented regions and populations, to combine data into even larger, standardized datasets to generate meaningful and globally relevant results.^8^ A collaborative mindset is indispensable to sharing expertise internationally and facilitating new research opportunities, which will eventually enable the development of personalized therapies.

## Supplementary Material

Data S1

## Figures and Tables

**TABLE 1:** Demographic, clinical, and genetic data of the entire GP2’s MN cohort compared with The MJFF GMPD cohort and all individuals with parkinsonism from both efforts with reported genetic findings

Demographic, Clinical, and Genetic Variables	GP2’s MN Cohort (n = 4824)		MJFF GMPD Cohort (n = 3185)		Individuals from MJFF GMPD and GP2’s MN with Reported Genetic Findings (n = 539)	
	Available Data	Missing Data	Available Data	Missing Data	Available Data	Missing Data
Sex, n (%)		150 (3.1%)		0 (0%)		0 (0%)
Female	2003 (42.9%)		1491 (46.8%)		268 (49.7%)	
Male	2671 (57.1%)		1694 (53.2%)		271 (50.3%)	
Age, median (IQR), y	65 (55–74.5)	307 (6.4%)	63 (51–71)	244 (7.7%)	63 (52–74)	18 (3.3%)
AAO, median (IQR), y	49 (41–62)	816 (16.9%)	53 (42–63)	0 (0%)	51 (38.5–64)	36 (6.7%)
EOPD, n (%)	2169 (54.1%)		1367 (42.9%)		226 (44.9%)	
Non-EOPD, n (%)	1839 (45.9%)		1818 (57.1%)		277 (55.1%)	
Ancestry, n (%)		1124 (23.3%)		67 (2.1%)		100 (18.6%)
Native Hawaiian/Pacific Islander	1 (<0.1%)		4 (0.1%)		0 (0%)	
American Indian/Alaska Native	2 (0.1%)		1 (<0.1%)		0 (0%)	
Ashkenazi Jewish	16 (0.4%)		484 (15.5%)		1 (0.2%)	
Black/African American	28 (0.8%)		19 (0.6%)		5 (1.1%)	
Hispanic/Latino	72 (1.9%)		63 (2.0%)		4 (0.9%)	
Arab	104 (2.8%)		14 (0.4%)		2 (0.5%)	
White/European	1657 (44.8%)		2369 (76.0%)		341 (77.7%)	
Asian	1762 (47.6%)		150 (4.8%)		81 (18.5%)	
Other	58 (1.6%)		14 (0.4%)		5 (1.1%)	
Family history of parkinsonism, n (%)		1103 (22.9%)		672 (21.1%)		30 (5.8%)
Positive	2199 (59.1%)		1152 (45.8%)		277 (54.4%)	
Negative	1522 (40.9%)		1361 (54.2%)		232 (45.6%)	
Consanguinity, n (%)		2656 (55.1%)	n.a.	n.a.		342 (63.5%)
Yes	151 (7.0%)				7 (3.6%)	
No	2017 (93.0%)				190 (96.4%)	
Reported genetic findings, n (%)^a^		n.a.^b^		0 (0%)		0 (0%)
GBA1	279 (51.7%)		1224 (38.4%)		279 (51.7%)	
LRRK2	130 (24.1%)		1306 (41.0%)		130 (24.1%)	
SNCA	19 (3.5%)		115 (3.6%)		19 (3.5%)	
VPS35	2 (0.4%)		23 (0.7%)		2 (0.4%)	
PINK1	25 (4.6%)		75 (2.4%)		25 (4.6%)	
PRKN	79 (14.7%)		429 (13.5%)		79 (14.7%)	
PARK7/DJ1	5 (0.9%)		13 (0.4%)		5 (0.9%)	

a The genetic results originate from prior analyses of the various participating centers and not from the current analyses of the GP2’s MN. Genetic analyses within GP2 are still ongoing. Of the 539 affected individuals with reported genetic findings, 293 individuals were transferred from The MJFF GMPD project to the GP2’s MN, and 246 samples with reported genetic findings were submitted directly to GP2.

b Data are not yet available because genetic analyses of all GP2’s MN samples are still ongoing.

Abbreviations: GP2’s MN, Global Parkinson’s Genetics Program’s Monogenic Network; MJFF GMPD, The Michael J. Fox Foundation Global Monogenic PD; IQR, interquartile range; AAO, age at onset; EOPD, early-onset Parkinson’s disease (age at disease onset ≤ 50 years); non-EOPD, non-early-onset Parkinson’s disease (age at disease onset > 50 years); n.a., not available; PD, Parkinson’s disease.

## Data Availability

GP2 partnered with the online cloud computing platform Accelerating Medicines Partnership—Parkinson’s Disease (AMP PD; https://amp-pd.org) to share data generated by GP2. Anonymized data can be shared upon request and qualified researchers are encouraged to apply for direct access to the data through AMP PD.
